# Supplementary material for: Discovery of epi-Enprioline as a Novel Drug for the Treatment of Vincristine Resistant Neuroblastoma
Source: Int J Mol Sci. 2020 Sep 8;21(18):6577. doi: 10.3390/ijms21186577 (PMC7556009; doi:10.3390/ijms21186577)
Supplement: Supplementary file 1 [file ijms-21-06577-s001.zip › supplementary-for conversion/ijms-896429-supplementary.pdf]

A

Crystal data for compound (*rac*)-*epi*-enpiroline[a]

Chemical formula	$C_{19}H_{18}F_6N_2O$	
Formula Mass	404.35	
Crystal size/mm^3	0.15 × 0.05 × 0.05	
Crystal habit	White, needles	
Crystal system	Monoclinic	
Unit cell dimensions	a = 14.0135(10) Å	
	b = 17.1367(17) Å	β = 104.041(6) °
	c = 8.2317(5) Å	
Unit cell volume/Å^3	1917.7 (3)	
Collection temperature/K	293(2)	
Space group	$P2_1/c$	
Number of formula units per cell, Z	1	
Radiation type	MoKα	
Absorption coefficient, μ/mm^{-1}	0.127	
No. reflections measured	9126	
No. independent reflections	4423	
R_{int}	0.045	
Final R_1 values *($I>2\sigma(I)$)*	0.0782	
Final R_1 values *(all data)*	0.1551	
Final $wR(F^2)$ values *($I>2\sigma(I)$)*	1.02	
Goodness of fit on F^2	1.016	
Flack parameter		
Largest diff. peak and hole (e$^-$/Å^3)	0.155 and -0.221	
CCDC	1589743	

a) Both CF_3 groups were modeled as disordered with a relative occupation of 66:35 for the 4-phenyl- and 62:38 for the 2-pyridyl group.

B

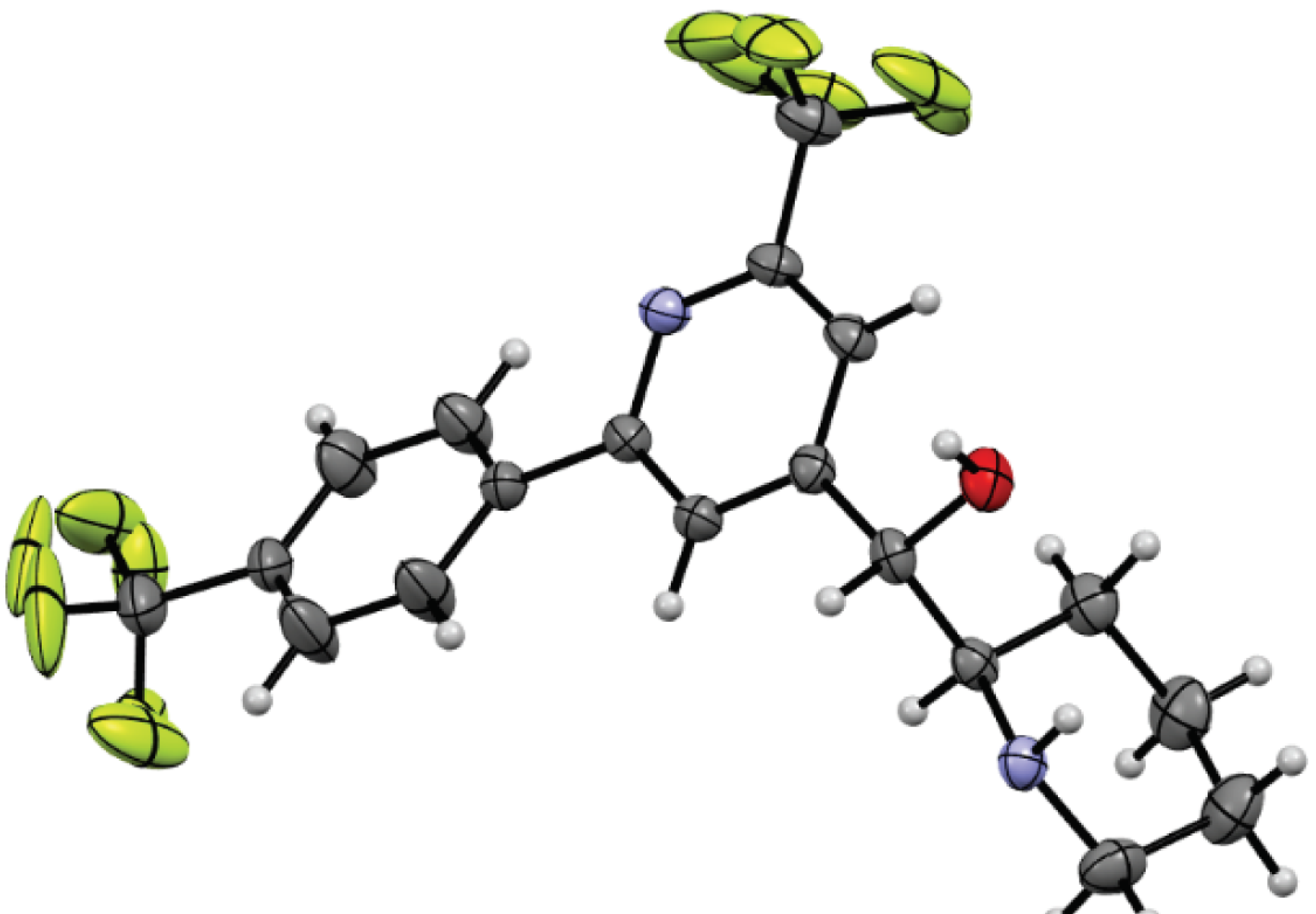

Supplementary Fig. 1

(A) The scXRD data for (rac)-epi-enpiroline. Single crystals were picked directly from the sample provided in the screening library. (B) The asymmetric unit of (rac)-epi-enpiroline. Thermal ellipsoids are shown at 30% probability. Gray = carbon atom; blue = nitrogen atom; red = oxygen atom; white = hydrogen atom (lower panel).

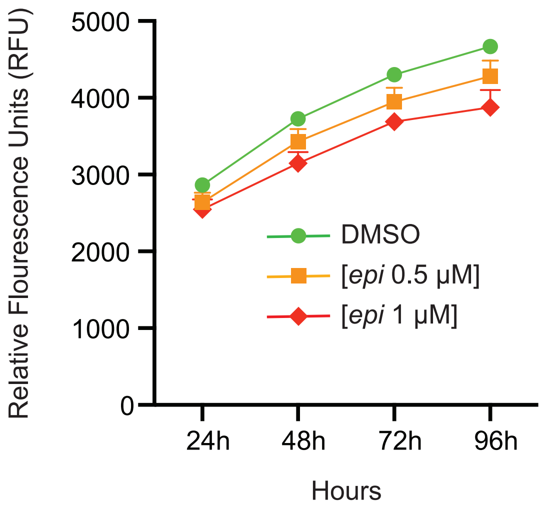

Relative Flourescence Units (RFU)
5000
4000
3000
2000
1000
0
24h
48h
72h
96h
Hours
DMSO
[*epi* 0.5 μM]
[*epi* 1 μM]

Supplementary Fig. 2

The viability of MEF cells was determined using Alamar blue fluorometric based assay. Cells were seeded in 96-well plates in triplicates and following incubation of cells (100µl of culture medium), 10% v/v Alamar blue (10µl) was added and the relative fluorescence units (RFU) was measured (excitation 530 nm, emission 590 nm) at 3h.

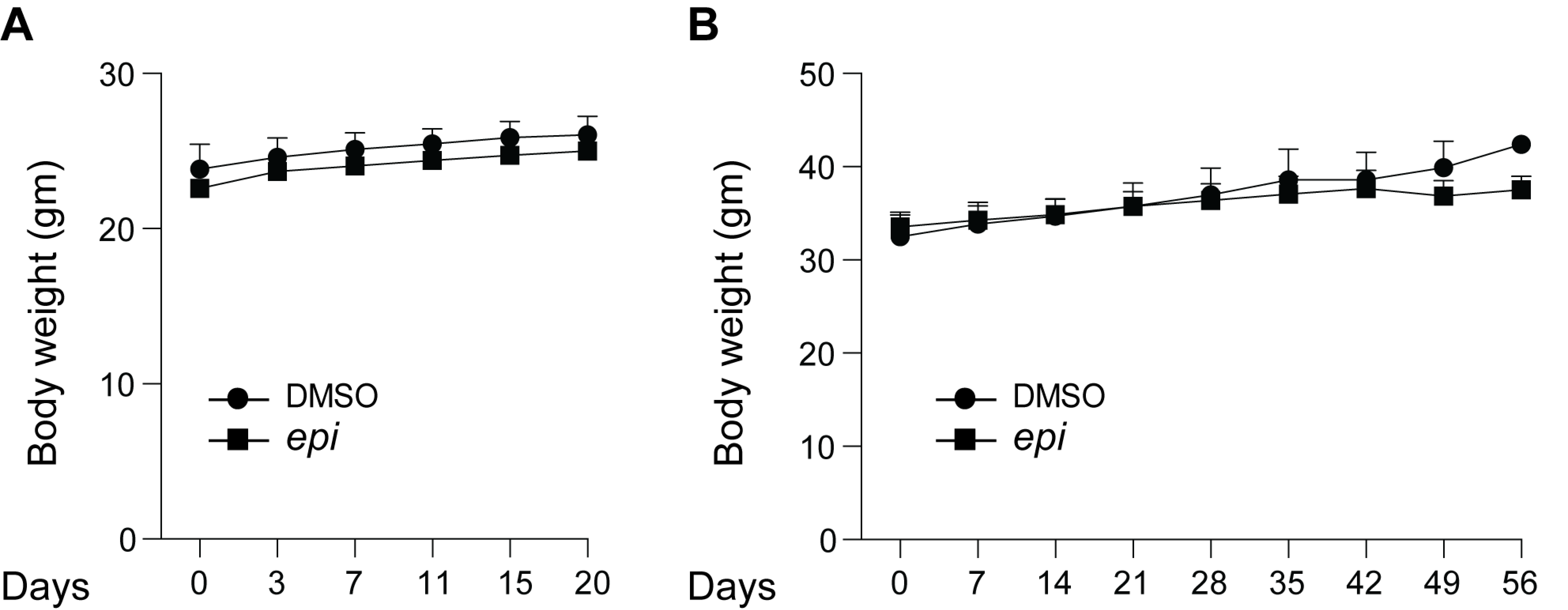

A
B
Body weight (gm)
30
20
10
0
Days
0
3
7
11
15
20
DMSO
epi
50
40
30
20
10
0
Days
0
7
14
21
28
35
42
49
56
DMSO
epi

Supplementary Fig. 3

Change in the total body weight (gram) of orthotopic-tumor bearing mice over the course of treatment period in mice that tumor cells including Be2C-VCR20 GFP-Luc (A) or LU-NB-2 GFP-Luc (B) were orthotopic implanted into adrenal gland and treated intraperitoneal with DMSO or *epi*-enprioline. The onset of treatment is counted as Day 0.

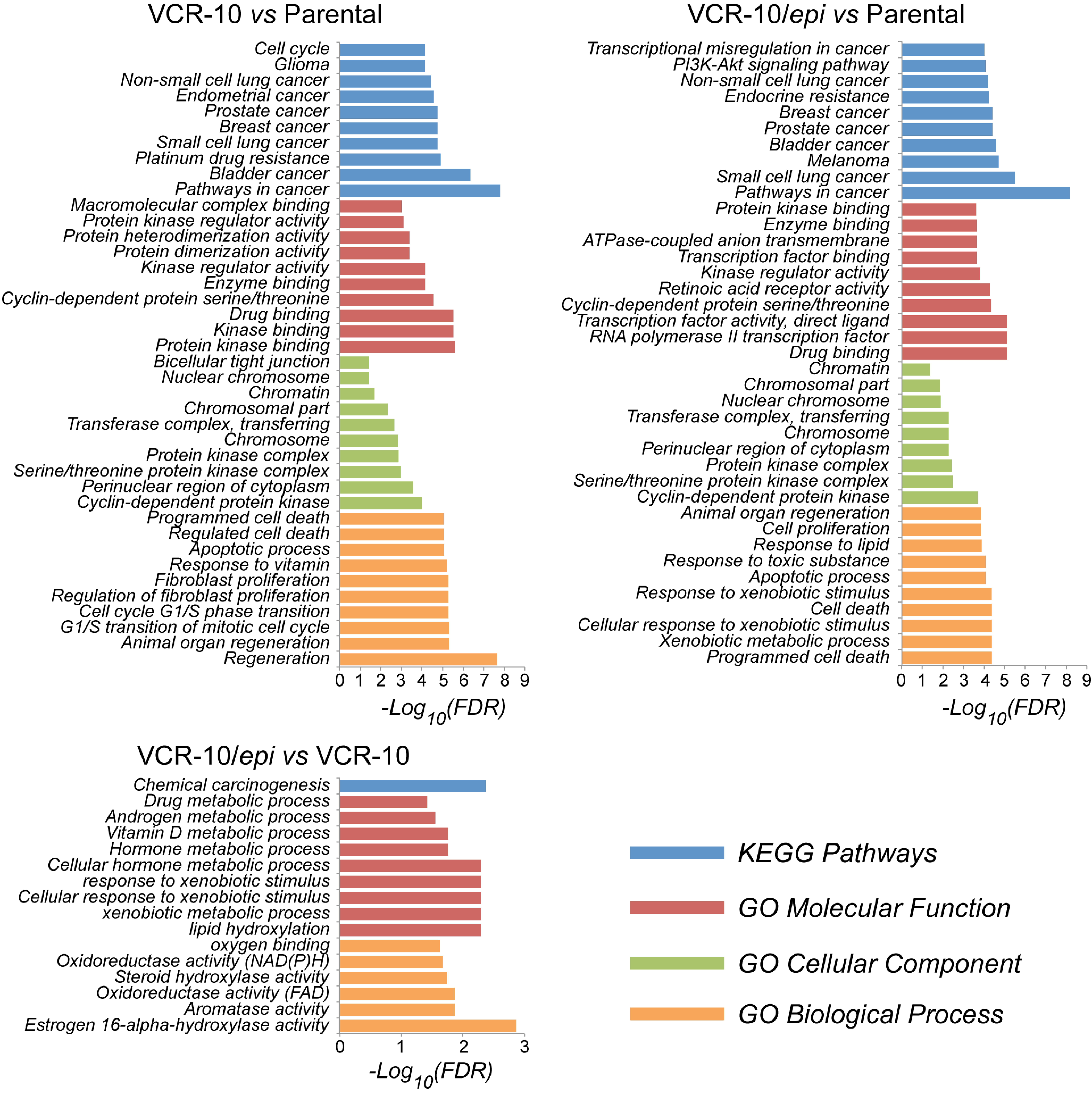

VCR-10 vs Parental
Cell cycle
Glioma
Non-small cell lung cancer
Endometrial cancer
Prostate cancer
Breast cancer
Small cell lung cancer
Platinum drug resistance
Bladder cancer
Pathways in cancer
Macromolecular complex binding
Protein kinase regulator activity
Protein heterodimerization activity
Protein dimerization activity
Kinase regulator activity
Enzyme binding
Cyclin-dependent protein serine/threonine
Drug binding
Kinase binding
Protein kinase binding
Bicellular tight junction
Nuclear chromosome
Chromatin
Chromosomal part
Transferase complex, transferring
Chromosome
Protein kinase complex
Serine/threonine protein kinase complex
Perinuclear region of cytoplasm
Cyclin-dependent protein kinase
Programmed cell death
Regulated cell death
Apoptotic process
Response to vitamin
Fibroblast proliferation
Regulation of fibroblast proliferation
Cell cycle G1/S phase transition
G1/S transition of mitotic cell cycle
Animal organ regeneration
Regeneration
0 1 2 3 4 5 6 7 8 9
-Log10(FDR)
VCR-10/epi vs Parental
Transcriptional misregulation in cancer
PI3K-Akt signaling pathway
Non-small cell lung cancer
Endocrine resistance
Breast cancer
Prostate cancer
Bladder cancer
Melanoma
Small cell lung cancer
Pathways in cancer
Protein kinase binding
Enzyme binding
ATPase-coupled anion transmembrane
Transcription factor binding
Kinase regulator activity
Retinoic acid receptor activity
Cyclin-dependent protein serine/threonine
Transcription factor activity, direct ligand
RNA polymerase II transcription factor
Drug binding
Chromatin
Chromosomal part
Nuclear chromosome
Transferase complex, transferring
Chromosome
Perinuclear region of cytoplasm
Protein kinase complex
Serine/threonine protein kinase complex
Cyclin-dependent protein kinase
Animal organ regeneration
Cell proliferation
Response to lipid
Response to toxic substance
Apoptotic process
Response to xenobiotic stimulus
Cell death
Cellular response to xenobiotic stimulus
Xenobiotic metabolic process
Programmed cell death
0 1 2 3 4 5 6 7 8 9
-Log10(FDR)
VCR-10/epi vs VCR-10
Chemical carcinogenesis
Drug metabolic process
Androgen metabolic process
Vitamin D metabolic process
Hormone metabolic process
Cellular hormone metabolic process
response to xenobiotic stimulus
Cellular response to xenobiotic stimulus
xenobiotic metabolic process
lipid hydroxylation
oxygen binding
Oxidoreductase activity (NAD(P)H)
Steroid hydroxylase activity
Oxidoreductase activity (FAD)
Aromatase activity
Estrogen 16-alpha-hydroxylase activity
0 1 2 3
-Log10(FDR)
KEGG Pathways
GO Molecular Function
GO Cellular Component
GO Biological Process

Supplementary Fig. 4

Gene Ontology and Pathway enrichment Top10. WebGestalt tool was queried with gene lists from the following contrasts: (i) Be2c-VCR vs Be2c-Control; (ii) Be2c-VCR-A7 vs Be2c-Control and (iii) Be2c-VCR-A7 vs Be2c-VCR. The search for enrichment was performed for GO categories (Molecular Function, Cellular Component and Biological Process) and KEGG Pathways. Significance level was set at FDR ≤ 0.05. Of the significantly enriched GO terms and pathways we show only the top 10 with lowest FDR.
